# Construction of a dynamic prediction model for diabetic retinopathy risk in adults with adult-onset type 1 diabetes: a real-world longitudinal cohort study

**DOI:** 10.3389/fcdhc.2026.1873754

**Published:** 2026-07-28

**Authors:** Di Bao, Yingying Li, Chun Mu, Qiuling Xing

**Affiliations:** 1 Department of Infection Management, National Health Commission Key Laboratory of Hormones and Development, Tianjin Key Laboratory of Metabolic Diseases, Tianjin Medical University Chu Hsien-I Memorial Hospital & Tianjin Institute of Endocrinology, Tianjin, China; 2 Tianjin Medical University, Tianjin, China; 3 Department of Ophthalmology, National Health Commission Key Laboratory of Hormones and Development, Tianjin Key Laboratory of Metabolic Diseases, Tianjin Medical University Chu Hsien-I Memorial Hospital & Tianjin Institute of Endocrinology, Tianjin, China; 4 Department of Medical Record, National Health Commission Key Laboratory of Hormones and Development, Tianjin Key Laboratory of Metabolic Diseases, Tianjin Medical University Chu Hsien-I Memorial Hospital & Tianjin Institute of Endocrinology, Tianjin, China

**Keywords:** adult-onset, diabetic retinopathy, dynamic prediction, nomogram, time-varying covariates, type 1 diabetes mellitus

## Abstract

**Objective:**

To construct and validate a dynamic prediction model for diabetic retinopathy (DR) risk in adults with adult-onset type 1 diabetes mellitus (T1DM).

**Methods:**

A longitudinal cohort of 572 adult-onset T1DM patients from a tertiary hospital in Tianjin, China (2014–2023) was analyzed. LASSO regression and multivariable Cox regression with time-varying covariates (duration and HbA1c) were used to identify independent predictors of DR and to construct a nomogram. Model performance was assessed using the concordance index (C-index), area under the receiver operating characteristic curve (AUC), Brier score, calibration curves, Hosmer-Lemeshow test, and decision curve analysis (DCA). Internal validation was performed by bootstrap resampling (1000 replicates).

**Results:**

Multivariable Cox regression identified diabetic peripheral vascular disease, diabetic kidney disease, metabolic bone disease, dynamic disease duration, time-varying HbA1c, body mass index (BMI), and gender as independent predictors of DR. The dynamic model had a C-index of 0.704 (corrected: 0.681), with 1-, 2-, and 3-year AUCs of 0.704, 0.713, and 0.732, respectively. Brier scores were all <0.1. Calibration was acceptable at 2 and 3 years; the 1-year calibration slope was elevated (1.895). Hosmer-Lemeshow test P-values were >0.05 at all time points. The dynamic model outperformed a traditional static model in both discrimination and calibration. DCA showed favorable net clinical benefit for 2- and 3-year predictions.

**Conclusion:**

This study demonstrates the feasibility of dynamic DR risk assessment in adult-onset T1DM. The proposed nomogram may serve as an auxiliary tool for predicting 2- to 3-year DR risk, supporting nurses in risk stratification and individualized screening guidance.

## 1 Introduction

Diabetic retinopathy (DR) is a leading cause of blindness among working-age adults. Early identification of high-risk patients and initiation of regular screening are critical for timely detection and prevention of progression to vision-threatening stages (1). In China, adult-onset type 1 diabetes mellitus (T1DM), including latent autoimmune diabetes in adults (LADA), accounts for over 65% of new T1DM cases (2), representing a large population with distinct clinical characteristics. Compared to type 2 diabetes mellitus (T2DM), this group has a higher prevalence and faster progression of DR (3), posing greater challenges for DR risk management.

National and international guidelines have highlighted the crucial role of screening education and risk-stratified dynamic monitoring in the prevention and management of DR (4, 5). Diabetes specialist nurses, as core members of the multidisciplinary team responsible for delivering Diabetes Self-Management Education and Support (DSMES), assume multiple roles—including assessment, education, and coordination—in the care of individuals with T1DM (6, 7).

However, there is a lack of DR risk prediction tools specifically developed for adult-onset T1DM in China. Existing models are mostly built on T2DM or juvenile-onset T1DM populations and often treat disease duration and HbA1c as baseline fixed variables (8, 9). This “static” approach fails to capture the dynamic nature of DR risk that accumulates over time and fluctuates with glycemic control. Moreover, it cannot meet the needs of individualized, continuous risk management in nursing practice, making it difficult for nurses to accurately identify high-risk individuals and conduct effective risk communication and follow-up guidance.

Therefore, this study leveraged real-world longitudinal survival data to develop a dynamic prediction model for DR risk in the adult-onset T1DM population, aiming to provide a reference for nurses to implement individualized DSMES and to optimize screening and follow-up strategies.

## 2 Methods

### 2.1 Study population

We retrospectively collected data from the electronic medical record system of a tertiary hospital in Tianjin, China, between January 2014 and December 2023. Inclusion criteria were: ① diagnosis of T1DM confirmed by ICD-10 coding and clinical features (e.g., acute symptoms, insulin dependence), and evidence of positive islet autoantibodies (e.g., GAD-Ab) and/or low or progressively declining C-peptide levels (7); ② age at diagnosis ≥18 years; ③ at least two valid fundus examination records. Exclusion criteria were: ① DR present at baseline; ② opaque ocular media; ③ missing key data (e.g., HbA1c, duration). Sample size was estimated based on the ‘10 events per variable’ (EPV) rule. As no local longitudinal data were available to project the expected cumulative incidence of DR during follow-up, we derived the expected event proportion from a published meta-analysis of DR prevalence in the Chinese diabetic population, which reported a pooled prevalence of 22.4% (10). This estimate served as a conservative proxy for the expected event proportion over the planned follow-up period. Assuming 10 candidate predictors and a data missing rate of approximately 10%, the required sample size was at least 497 patients. The study was approved by the ethics committee, which granted a waiver of informed consent (approval No. ZXYJNYYsKMEC2024-41).

### 2.2 Follow-up and outcome definition

The start date was the date of first meeting inclusion criteria. Follow-up ended at the occurrence of DR (any new diagnosis of DR) or the last recorded date (no deaths occurred during the study). Fundus examinations were performed using a non-mydriatic (undilated) retinal camera (Topcon TRC; Topcon, Japan). For each eye, 45°color fundus photographs were obtained, centered on both the macula and the optic disc. The images were captured by trained ophthalmic technicians and independently interpreted by two or more experienced attending ophthalmologists according to the Chinese Clinical Practice Guideline for Diabetic Retinopathy (2022) (4). In cases of disagreement, a final decision was reached by consensus with a senior ophthalmologist. In this study, DR was diagnosed if either eye met the diagnostic criteria for diabetic retinopathy according to the above guideline.

#### 2.2.1 Data Collection

The following data were collected from the electronic medical record system:

- General information: sex, age, body mass index (BMI), systolic and diastolic blood pressure, smoking status, and alcohol consumption.
- Clinical data: age at diabetes diagnosis, disease duration, diabetes-related complications, and comorbidities.
- Laboratory and examination indicators: HbA1c, liver and kidney function tests, lipid profile, routine blood and urine tests, and fundus examination results.

#### 2.2.2 Variable Definitions

- Time-varying covariates:

*Dynamic disease duration*: defined as the cumulative time from T1DM diagnosis to each follow-up visit, calculated as (current clinical record date – diabetes diagnosis date)/365.25 (years).

*Time-varying HbA1c*: defined as the most recent measured HbA1c value prior to each follow-up visit (i.e., the value closest in time to the visit date), which reflects the patient’s recent glycemic control status. For statistical analysis, we adopted the Last Observation Carried Forward (LOCF) approach: the latest available test value at each time point was utilized. If no new laboratory record was available at a given follow-up, the measurement from the prior visit was carried forward until it was replaced by a subsequent new test result.

- Metabolic Bone Disease: defined according to the Chinese Guidelines for the Diagnosis and Treatment of Primary Osteoporosis (2022) (11).

### 2.3 Statistical analysis

Data were analyzed using R version 4.5.1. Continuous variables were expressed as mean ± standard deviation (SD) or median (interquartile range, IQR), with between-group comparisons using t-test or Wilcoxon rank-sum test. Categorical variables were expressed as n (%) and compared using χ² test or Fisher’s exact test. Multiple imputation (m=5) was used for missing values. Least absolute shrinkage and selection operator (LASSO) regression with 10-fold cross-validation was used for variable selection, followed by multivariable Cox regression. The ‘tmerge’ function was used to restructure longitudinal data into counting process format to handle time-varying covariates. Model performance was assessed using C-index, time-dependent area under the receiver operating characteristic curve (AUC), Brier score, calibration curves, Hosmer-Lemeshow test, and decision curve analysis. Internal validation was performed using bootstrap with 1000 resamples. A two-sided P<0.05 was considered statistically significant.

## 3 Results

### 3.1 Baseline characteristics

A total of 572 adult-onset T1DM patients without DR at baseline were included, of whom 348 (60.8%) had LADA. The cohort included 283 females (49.5%), mean age 45.42 ± 14.03 years. The median follow-up duration was 4.02 (2.07, 6.84) years, and the median HbA1c testing interval was 1.90 (1.38, 2.55) quarters, detailed follow-up data are presented in **Supplementary Table 1**. Cumulative DR incidence was 37.4% (214/572). The actual number of outcome events substantially surpassed the minimum EPV requirement of 10. Far from indicating an insufficient sample size, higher-than-expected event counts actually enhanced the statistical power of our multivariable analyses, providing more reliable parameter estimates than originally planned. Baseline comparisons between DR and non-DR groups are shown in **Table 1**.

**Table 1:** Baseline characteristics of patients with and without diabetic retinopathy (DR).

Characteristics	Non-DR group (n=358)	DR group (n=214)	P-value
Type of diabetes			0.458
Classical T1DM	136 (38.0%)	88 (41.1%)	
LADA	222 (62.0%)	126 (58.9%)	
Sex			0.001
Male	162 (45.3%)	127 (59.3%)	
Female	196 (54.7%)	87 (40.7%)	
Age (years)	45.55 ± 14.86	45.20 ± 12.56	0.761
BMI (kg/m²)	21.52 ± 3.32	22.16 ± 3.18	0.025
SBP (mmHg)	117.78 ± 14.84	118.93 ± 16.14	0.386
DBP (mmHg)	74.02 ± 9.27	74.99 ± 10.06	0.242
Onset age (years)	42.13 ± 14.26	38.56 ± 11.67	0.001
Baseline duration (years)	1.25 (0.00, 4.00)	5.00 (2.00, 10.00)	<0.001
Baseline HbA1c (%)	8.56 ± 2.38	9.28 ± 2.37	0.022
Family history of diabetes (Yes)	160 (44.7%)	85 (39.7%)	0.282
Diabetic kidney disease (Yes)	10 (2.8%)	48 (22.4%)	<0.001
Diabetic peripheral neuropathy (Yes)	54 (15.1%)	29 (13.6%)	0.703
Diabetic peripheral vascular disease (Yes)	45 (12.6%)	44 (20.6%)	0.015
Insulin use (Yes)	266 (74.3%)	182 (85.0%)	0.003
History of hypertension (Yes)	50 (14.0%)	33 (15.4%)	0.722
History of cardiovascular disease (Yes)	75 (20.9%)	68 (31.8%)	0.005
Bone metabolic disorders (Yes)	63 (17.6%)	55 (25.7%)	0.020
Smoking (Yes)	143 (39.9%)	102 (47.7%)	0.086
Alcohol use (Yes)	98 (27.4%)	78 (36.4%)	0.029
Tg (mmol/L)	0.85 (0.63, 1.16)	0.89 (0.67, 1.23)	0.101
TC (mmol/L)	4.75 ± 1.16	4.67 ± 0.99	0.455
HDL-C (mmol/L)	1.43 ± 0.39	1.44 ± 0.35	0.846
LDL-C (mmol/L)	2.98 ± 0.91	2.86 ± 0.79	0.136
Scr (μmol/L)	56.98 ± 13.47	58.41 ± 13.32	0.246
SUA (μmol/L)	225.61 ± 68.52	242.68 ± 73.10	0.009
AST (U/L)	17.25 (14.13, 22.28)	16.90 (14.10, 22.20)	0.793
ALT (U/L)	15.40 (11.80, 22.10)	15.70 (12.30, 22.40)	0.496
GGT (U/L)	13.50 (10.85, 19.15)	14.50 (11.80, 22.55)	0.008
WBC (×10^9^/L)	5.94 ± 1.80	6.12 ± 2.01	0.252
Hb (g/L)	135.48 ± 17.27	136.99 ± 15.36	0.305
PLR	123.70 ± 53.40	125.73 ± 58.07	0.667
NLR	1.70 (1.20, 2.30)	1.75 (1.20, 2.50)	0.492

Data are expressed as the mean ± SD or the median (Q1, Q3) for continuous variables, where Q1 and Q3 denote the 25th and 75th percentiles, respectively; categorical variables are expressed as n (%).

LADA, latent autoimmune diabetes in adults; BMI, body mass index; HbA1c, glycated hemoglobin; SBP, systolic blood pressure; DBP, diastolic blood pressure; Tg, triglyceride; TC, total cholesterol; Scr, serum creatinine; SUA, serum uric acid; BUN, blood urea nitrogen; HDL-C, high-density lipoprotein cholesterol; LDL-C, low-density lipoprotein cholesterol; AST, aspartate aminotransferase; ALT, alanine aminotransferase; GGT, gamma-glutamyl transferase; WBC, White blood cell; Hb, hemoglobin; PLR, Platelet-to-lymphocyte ratio; NLR, Neutrophil-to-lymphocyte ratio.

### 3.2 Variable selection and model construction

**Figure 1:**
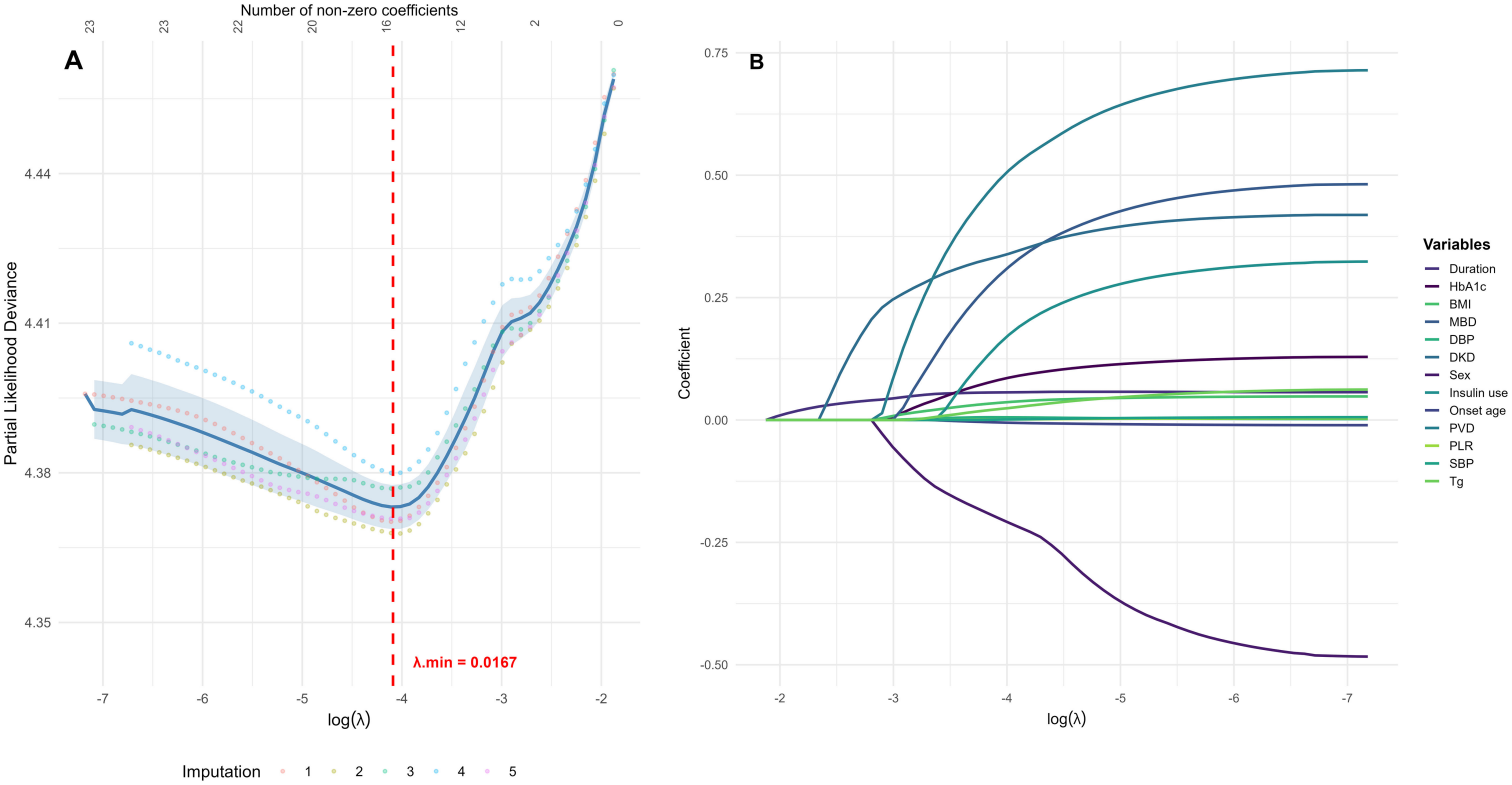
LASSO regression for the screening of the predictor variables (Averaged over 5 Imputed Datasets). **(A)** LASSO regression cross-validation plot. **(B)** LASSO regression coefficient path plot. HbA1c: time-varying HbA1c, reflecting the most recent glycemic control at each follow-up visit; Duration: dynamic disease duration, reflecting cumulative duration from diagnosis to current visit; BMI, body mass index; MBD, metabolic bone disease; DKD, diabetic kidney disease; PVD, diabetic peripheral vascular disease; SBP, systolic blood pressure; DBP, diastolic blood pressure; Tg, triglyceride; PLR, platelet-to-lymphocyte ratio.

**Figure 2:**
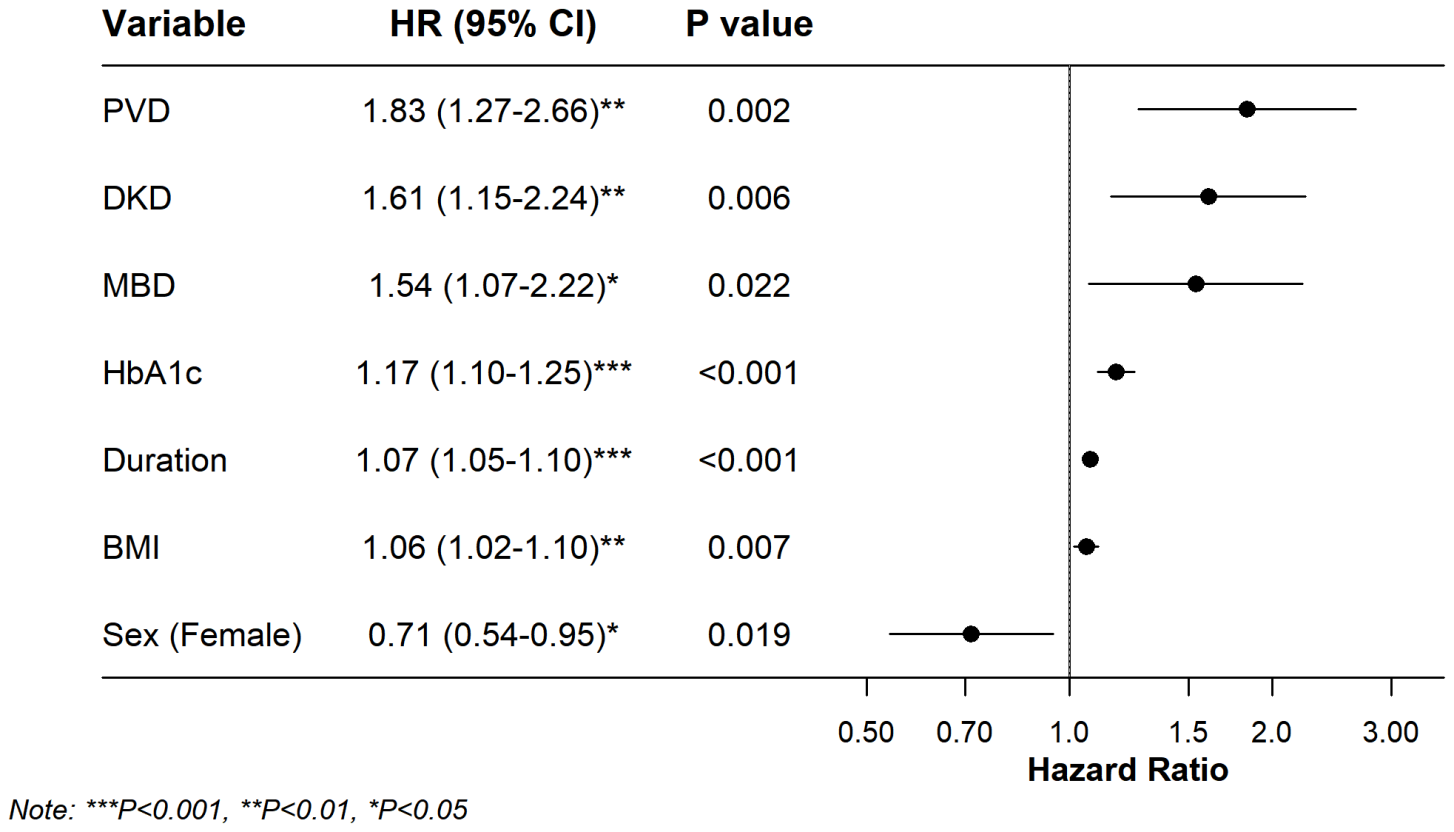
Forest plot of multivariable Cox regression for diabetic retinopathy in adult-onset T1DM. PVD, diabetic peripheral vascular disease; DKD, diabetic kidney disease; MBD, metabolic bone disease; BMI, body mass index; HbA1c: time-varying HbA1c, reflecting the most recent glycemic control at each follow-up visit; Duration: dynamic disease duration, reflecting cumulative duration from diagnosis to current visit.

LASSO regression with 10-fold cross-validation identified 13 predictors commonly selected across the five imputed datasets (**Figure 1**). Multivariable Cox regression retained seven independent predictors of DR (**Figure 2**), including peripheral vascular disease (HR = 1.83, 95% CI: 1.27-2.66), diabetic kidney disease (HR = 1.61, 95% CI: 1.15-2.24), metabolic bone disease (HR = 1.54, 95% CI: 1.07-2.22), dynamic disease duration (HR = 1.07, 95% CI: 1.05-1.10), time-varying HbA1c (HR = 1.17, 95% CI: 1.10-1.25), BMI (HR = 1.06, 95% CI: 1.02-1.10), and female sex (HR = 0.71, 95% CI: 0.54-0.95). These seven independent predictors were then used to construct the dynamic model.

### 3.3 Evaluation of the dynamic model

**Figure 3:**
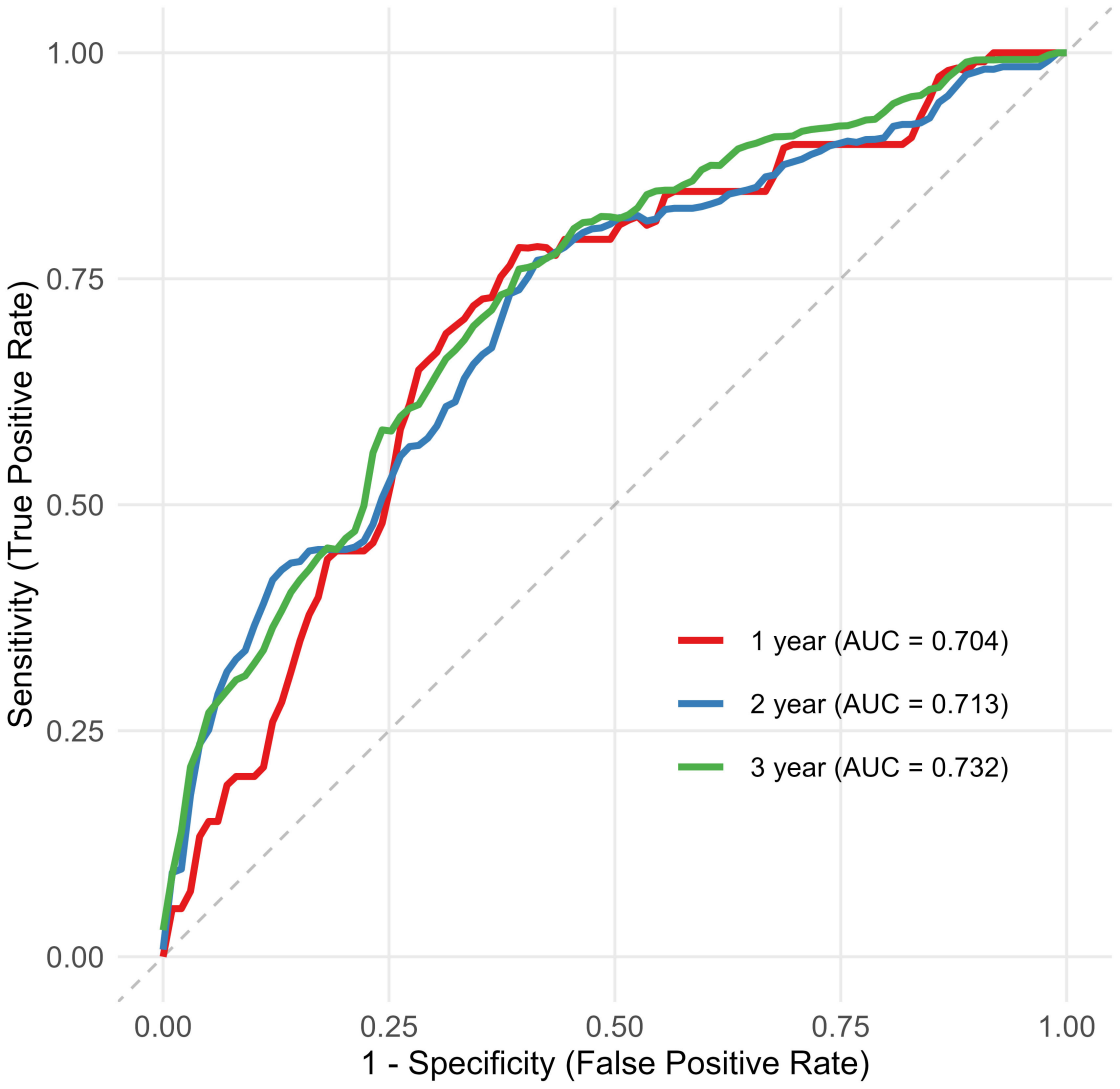
Time-dependent ROC curves of the dynamic model.

The dynamic model’s C-index was 0.704 (bootstrap-corrected: 0.681). Additionally, time-dependent receiver operating characteristic (ROC) curve analysis was applied to evaluate the accuracy of the dynamic model in predicting DR at different time points. The results showed the AUCs at 1-, 2-, and 3-year were 0.704, 0.713, and 0.732 (**Figure 3**), indicating moderate discrimination that improved with longer prediction horizons.

**Figure 4:**
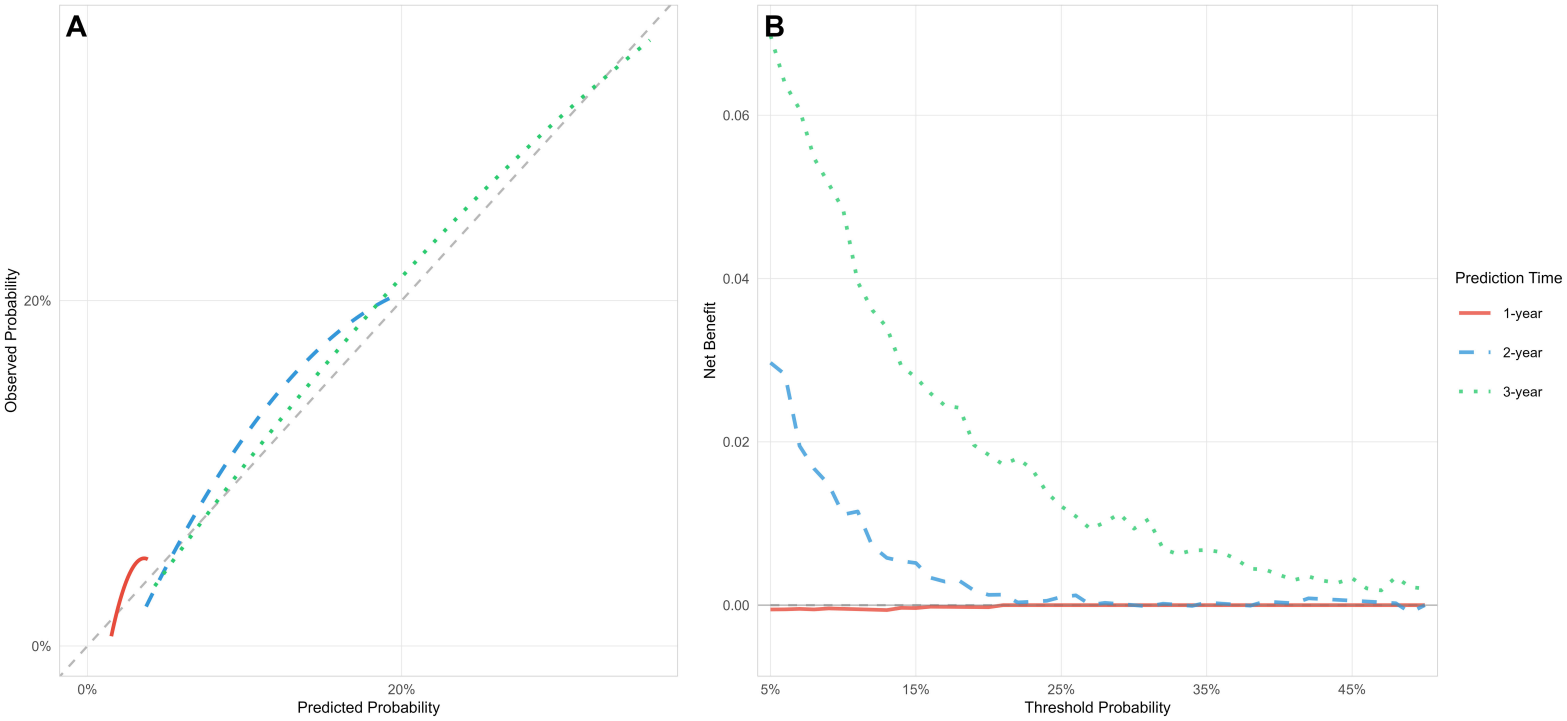
Calibration curves **(A)** and decision curve analysis **(B)** of the dynamic model at 1-, 2-, and 3-year for diabetic retinopathy in adult-onset T1DM.

Brier scores at 1-, 2-, and 3-year were all <0.1 (**Table 2**), indicating low overall prediction error. The 1-year calibration slope was elevated (1.895), while 2- and 3-year calibration curves were close to the diagonal (**Figure 4A**). Hosmer-Lemeshow test P-values were >0.05 at all time points (**Table 3**), suggesting no significant difference between predicted and observed probabilities.

**Table 2:** Performance comparison between dynamic and traditional static models.

Metric	Dynamic model (95% CI)	Static model (95% CI)	Improvement (%)
C-index	0.704 (0.703-0.705)	0.675 (0.673-0.675)	+4.30
1-year AUC	0.704 (0.698-0.708)	0.655 (0.652-0.657)	+7.50
2-year AUC	0.713 (0.712-0.714)	0.674 (0.673-0.675)	+5.80
3-year AUC	0.732 (0.728-0.735)	0.727 (0.725-0.727)	+0.70
1-year Brier score	0.020 (0.011-0.028)	0.035 (0.020-0.050)	-42.80
2-year Brier score	0.063 (0.050-0.076)	0.121 (0.094-0.147)	-47.90
3-year Brier score	0.093 (0.079-0.107)	0.194 (0.162-0.226)	-52.06

Brier score ranges 0–1, lower values indicate better accuracy.

Improvement = (static value – dynamic value)/static value × 100%.

**Table 3:** Comprehensive calibration metrics of the dynamic model.

Prediction time	Calibration slope (95% CI)	Mean absolute error	Hosmer-Lemeshow χ² (P)
1- year	1.895 (1.782-2.008)	0.0091	8.203 (0.426)
2- year	1.146 (1.072-1.220)	0.0175	8.589 (0.382)
3- year	1.008 (0.945-1.071)	0.0138	7.267 (0.514)

DCA (**Figure 4B**) showed that the model provided net clinical benefit for 2- and 3-year predictions across threshold probabilities of 5%–50%. Net benefit at 1 year was limited, likely due to the low 1-year DR incidence (2.0%) in this cohort.

To elucidate the added value of incorporating time-varying information for improving predictive performance, a traditional static Cox model was constructed using baseline HbA1c and baseline disease duration instead of time-varying covariates. The dynamic model outperformed the static model in C-index, AUC at all time points, and Brier scores (**Table 2**). Specifically, Brier score reductions were 42.8% at 1- year, 47.9% at 2- year, and 52.1% at 3- year.

Additionally, the generalizability and robustness of the dynamic model across different subtypes of adult-onset T1DM—classical T1DM (defined as adult-onset T1DM with acute symptomatic onset, ketosis-proneness, absolute insulin requirement within one year, and positivity for at least one islet autoantibody) and LADA—were evaluated using subgroup analysis and interaction tests. As shown in **Table 4**, no significant interactions were observed for any of the predictors between the subgroups (all P > 0.05), indicating that diabetes subtype did not significantly modify the association between the predictors and DR risk, thereby supporting the rationale for pooling the two subtypes in the model.

**Table 4:** Subgroup analysis and interaction tests for the dynamic model.

Variable	Classical T1DM		LADA		P-value for interaction
	HR(95% CI)	P-value	HR(95% CI)	P-value	
PVD	1.66 (0.87-3.17)	0.122	1.91 (1.17-3.12)	**0.010**	0.853
DKD	1.81 (1.02-3.20)	**0.042**	1.48 (0.95-2.31)	0.086	0.482
MBD	1.68 (0.92-3.08)	0.092	1.44 (0.88-2.34)	0.426	0.646
HbA1c	1.22 (1.10-1.36)	**<0.001**	1.14 (1.05-1.24)	**0.002**	0.244
Duration	1.07 (1.03-1.11)	**<0.001**	1.08 (1.05-1.11)	**<0.001**	0.673
BMI	1.12 (1.04-1.20)	**0.003**	1.02 (0.97-1.08)	0.426	0.057
Sex (female)	0.64 (0.40-1.03)	0.068	0.77 (0.54-1.11)	0.167	0.552

PVD, diabetic peripheral vascular disease; DKD, diabetic kidney disease; MBD, metabolic bone disease; BMI, body mass index; HbA1c: time-varying HbA1c, reflecting the most recent glycemic control at each follow-up visit; Duration: dynamic disease duration, reflecting cumulative duration from diagnosis to current visit. Bold values indicate P < 0.05.

**Figure 5:**
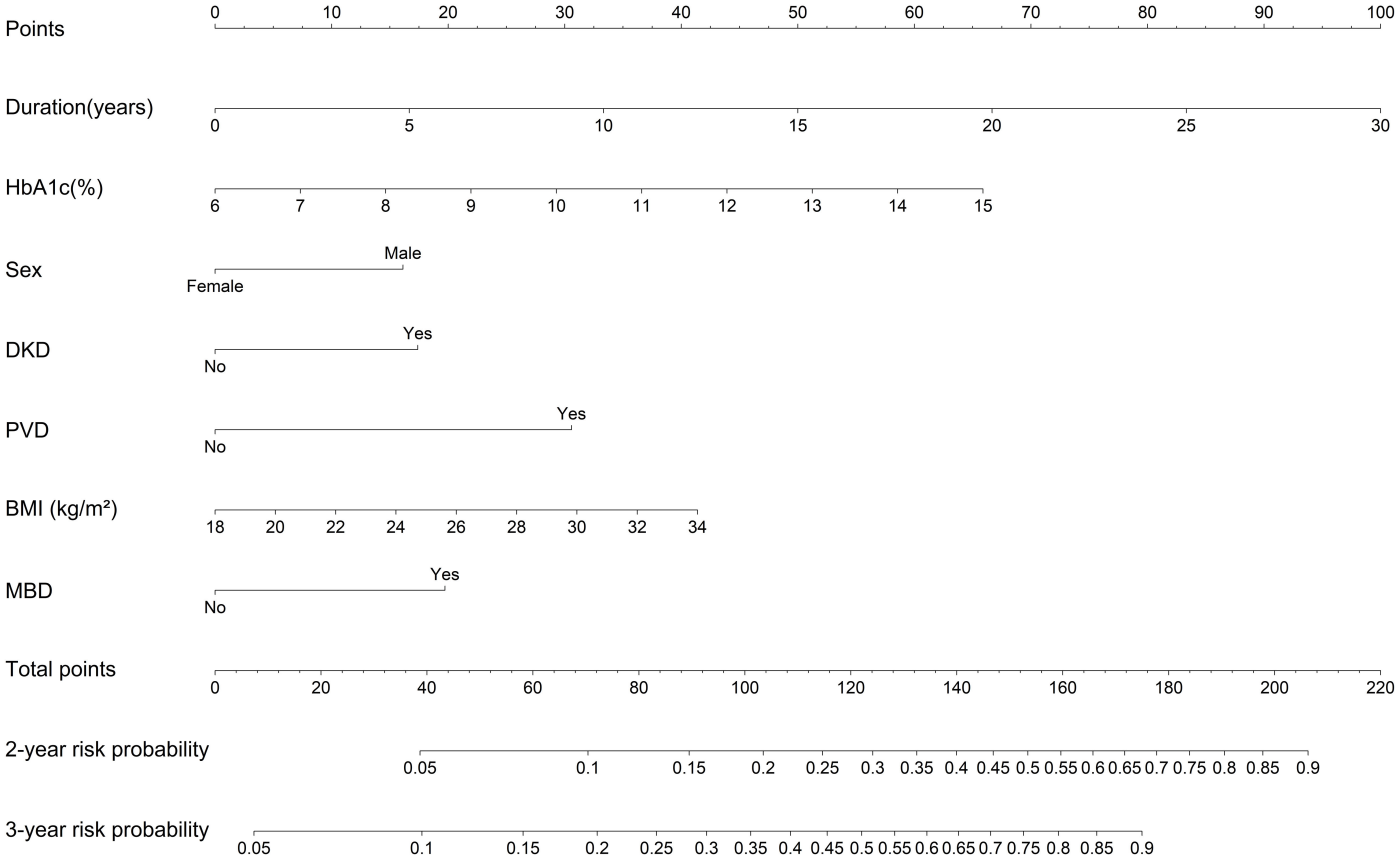
Nomogram for predicting the risk of diabetic retinopathy at 2- and 3- year in adult-onset T1DM. PVD, diabetic peripheral vascular disease; DKD, diabetic kidney disease; MBD, metabolic bone disease; BMI, body mass index; HbA1c: time-varying HbA1c, reflecting the most recent glycemic control at each follow-up visit; Duration: dynamic disease duration, reflecting cumulative duration from diagnosis to current visit.

Based on the above results, a nomogram was constructed (**Figure 5**). In clinical practice, real-time clinical data (current disease duration, most recent HbA1c level, presence of diabetic kidney disease/peripheral vascular disease/metabolic bone diseases, BMI and sex) are required to calculate the 2- to 3-year risk probability of diabetic retinopathy.

## 4 Discussion

### 4.1 Risk factors for DR in adult-onset T1DM

#### 4.1.1 Dynamic disease duration and time-varying HbA1c

Diabetes duration and glycemic control are well-established determinants of DR (4). The Diabetes Control and Complications Trial (DCCT) and its follow-up have confirmed that cumulative hyperglycemic exposure is the fundamental driver of microvascular complications in T1DM (12). Hirose et al. demonstrated the central role of cumulative glycemic exposure in DR development in T1DM (13). Unlike T2DM, the time of diagnosis in T1DM is relatively unambiguous, lending greater accuracy and reliability to disease duration as a time-varying variable. This makes “dynamic disease duration” particularly valuable for risk prediction in the T1DM population. By modelling both disease duration and HbA1c as time-varying covariates, our approach essentially quantifies the cumulative effects of time and glycaemic exposure. Both predictors were selected into the final model, collectively reflecting the core pathophysiological feature of DR development—cumulative damage—thereby aligning closely with the concept of “metabolic memory” and underscoring the continuity and dynamic nature of health management. Ju et al. (2024) found that cumulative fasting glucose load captured DR risk adequately (14), supporting our methodological choice. These findings suggest that, when managing adult-onset T1DM patients with acute disease onset and complex glycemic fluctuation patterns, nurses should interpret both prolongation of disease duration and each HbA1c measurement as immediate dynamic risk warning signals. By translating the abstract concept of “long-term control” into tangible emphasis on each follow-up HbA1c value, this approach enhances patients’ awareness of short-term treatment targets and fosters proactive behavioral adjustment.

#### 4.1.2 Diabetic kidney disease and diabetic peripheral vascular disease

Diabetic kidney disease (DKD) and diabetic peripheral vascular disease (PVD) both demonstrated strong associations with DR in our model, each through distinct pathophysiological pathways. As a classical microvascular complication, DKD shares with DR a well-recognised “reno-retinal axis,” characterised by common microvascular injury mechanisms—including activation of the Janus kinase/signal transducer and activator of transcription (JAK/STAT) pathway, inflammation, oxidative stress, and endothelial dysfunction (15). In contrast, PVD reflects systemic atherosclerotic burden and shares a broader set of risk factors with DR, including chronic hyperglycaemia, dyslipidaemia and systemic inflammation. Retinal microvasculature, as an integral component of the systemic vascular bed, is unlikely to remain unaffected in the presence of such widespread vascular damage. A prospective study (16) further demonstrated that patients with diabetic peripheral vascular disease had a significantly increased risk of developing microvascular complications, suggesting that PVD is not merely a local haemodynamic abnormality but rather a clinical manifestation of systemic vascular network impairment. Collectively, these lines of evidence may explain why both DKD and PVD emerged as strong predictors in our model.

Of note, although diabetic peripheral neuropathy (DPN) is also a well-recognised microvascular complication, it was not retained as an independent predictor in our final model. This finding is likely attributable to two factors. First, the prevalence of DPN showed only a minimal difference between the DR and non-DR groups in our cohort (13.6% vs. 15.1%, P = 0.703), which fundamentally limited its statistical discriminative capacity. Second, unlike DKD, the pathogenesis of DPN involves not only microvascular ischaemic damage but also non-vascular mechanisms, including neuronal metabolic toxicity, neurotrophic factor deficiency, and autoimmune-mediated inflammatory responses, which may result in an insufficiently direct pathogenic overlap with DR.

In summary, DKD and PVD, as indicators of the “renal-retinal axis” or “systemic vascular burden”, have a higher predictive value for DR risk in patients with adult-onset T1DM than DPN. Therefore, when patients with adult-onset T1DM are complicated by DKD or PVD, nurses should regard it as a clear indication to initiate or strengthen fundus screening, and actively coordinate multidisciplinary collaboration to promote early detection and timely intervention, thereby improving the long-term visual prognosis of this high-risk group.

#### 4.1.3 Metabolic bone diseases

Metabolic bone diseases were identified as an independent risk factor for DR. This association has been reported in T2DM: Chen et al. (2025) found that serum PINP (a bone formation marker) was inversely associated with DR risk (17). Possible mechanisms include vitamin D deficiency, insulin resistance, inflammation, and advanced glycation end-products. In T1DM, absolute insulin deficiency and autoimmune responses may affect bone health via microvascular damage to skeletal blood flow and bone remodeling (18). Although the mechanisms are not fully understood, bone health education may be considered in comprehensive management of adult-onset T1DM.

#### 4.1.4 BMI and sex

Studies have shown that BMI is closely associated with the development of DR in young and middle-aged diabetic patients (19). Although the mean BMI in our cohort was within the normal range, it remained independently associated with DR after adjusting for other factors, highlighting the unique importance of weight management for microvascular complication prevention in the specific population of adults with adult-onset T1DM. The underlying mechanism may be related to the phenomenon of “metabolically obese normal weight”. Some adults with adult-onset T1DM, particularly those with the LADA subtype, retain partial islet function; combined with the initiation of insulin therapy, this can lead to normal BMI but accumulated visceral fat. Visceral fat may drive the development of DR through mechanisms such as exacerbating vascular endothelial injury and promoting the release of inflammatory cytokines (20). This suggests that weight management in this population should go beyond simply achieving a “normal” BMI and pay greater attention to dynamic changes in BMI over time. Furthermore, our study showed that female patients had a lower risk of DR than males (HR = 0.71), which is consistent with previous reports (21). This finding may be attributable to the protective effects of estrogen on endothelial function and potentially more proactive health-seeking behaviors among women. Nonetheless, this observation can provide a differentiated reference for risk communication by sex in nursing practice, thereby motivating patients’ initiative in self-management.

### 4.2 Model innovation and performance

The core innovation of this study lies in incorporating “disease duration” and “HbA1c” as time-varying covariates into the model, allowing risk assessment to be updated in real time as the patient’s disease progresses and glycemic control changes. This aligns with the concept of “dynamic monitoring” advocated in the 2024 American Academy of Ophthalmology (AAO) guidelines (5). In terms of predictive performance, the model demonstrated moderate discrimination (C-index = 0.704), which is comparable to the AUC of 0.723 reported by Niu et al. (22) for a static prediction model. Potential reasons include: a relatively high proportion of LADA patients in the sample (60.8%)—although subgroup analysis showed no significant interaction, the pathophysiological heterogeneity between LADA and classic T1DM may still compromise the discriminative accuracy of the overall model; second, the model is based on routine clinical indicators and lacks more granular dynamic predictors such as glycemic variability and lifestyle factors; in addition, the median follow-up of 4.02 years is still insufficient to capture the long-term trajectory of DR risk.

Regarding calibration, the overall prediction error was small (Brier score <0.1), but the 1-year calibration slope deviated from 1.0 (1.895) and the net clinical benefit was limited. This is likely due to the low incidence of DR within the first year (only 2%) in this study: in a low-event-rate scenario, predicted probabilities shrink toward the mean event rate, and even with ideal discrimination, the “net benefit” for clinical decision-making is constrained by the absolute number of events. Therefore, this model is more suitable for predicting DR risk at ≥2 years. Compared with traditional static models, our dynamic model showed improved discrimination and calibration, with individual prediction deviations within 1.8% (mean absolute error ranging from 0.0091 to 0.0175). This indicates that the model can more accurately avoid over-screening of low-risk patients and under-screening of high-risk patients, which is of great significance for optimizing individual screening strategies.

In summary, this study preliminarily validates the feasibility of dynamic DR risk assessment in adult-onset T1DM patients, but the model’s performance needs to be further optimized through external validation and the integration of multi-source dynamic indicators.

### 4.3 Potential clinical application

Current Chinese guidelines recommend that patients with T1DM diagnosed after puberty undergo their first fundus screening within 5 years of diagnosis, followed by annual examinations thereafter (23). While this strategy ensures universal coverage, it does not account for individual variations in risk trajectories. Furthermore, patients with diabetes often have poor awareness of DR risk, resulting in low annual screening adherence (24). The nomogram developed in this study is not intended to replace guideline-recommended annual screening, but rather to serve as an auxiliary risk stratification tool for refine the current uniform screening strategy and provide a reference for implementing DSMES in nursing practice. Using routine clinical indicators, nurses can quickly quantify a patient’s risk of developing DR over the next 2–3 years, thereby enabling active, risk-stratified management. Compared with the standard guideline-based strategy, our model offers several potential applications. First, it can identify high-risk individuals earlier than the fixed 5-year screening threshold: for patients with poor glycaemic control and concomitant complications (e.g., diabetic kidney disease or peripheral vascular disease), the model may signal elevated risk well before 5 years, prompting timely intensification of education and earlier ophthalmology referral. Second, it may help avoid overscreening in persistently low-risk patients; for those with stable glycaemic control and no complications, the model provides objective, quantitative evidence to support shared decision-making regarding extension of screening intervals, thereby reducing unnecessary healthcare burden and improving patient acceptance. Third, the dynamic nature of the model allows screening recommendations to be updated at each visit based on the patient’s most recent clinical profile, rather than remaining static between annual follow-ups. This dynamic, personalised approach aligns with the shared decision-making philosophy advocated in the 2024 American Academy of Ophthalmology guidelines (5). By translating abstract risk into tangible, patient-specific estimates, the nomogram not only enhances the relevance and persuasiveness of patient education but also strengthens patients’ understanding of the necessity of screening and their motivation for behavioral change. Furthermore, clear quantitative risk estimates help nursing staff more precisely tailor educational plans, coordinate multidisciplinary follow-up, and dynamically evaluate intervention outcomes – thereby moving T1DM care toward a more individualized, continuous, and collaborative direction.

### 4.4 Limitations and future directions

This study has several limitations. First, it is a single-center retrospective study with a modest sample size and no external validation. The generalizability of the model needs to be further tested in larger, multicenter, prospective cohorts. Second, the model achieved only moderate discrimination. Future studies should incorporate multi-dimensional data such as continuous glucose monitoring (CGM) metrics, self-management behaviors, and lifestyle factors to improve its discriminative ability. Longer follow-up would also enhance model performance. Finally, embedding the model into electronic medical record systems could facilitate its clinical usability and real-time risk assessment.

## 5 Conclusion

In response to the practical needs of diabetes specialist nursing, this study preliminarily constructed a dynamic prediction nomogram for diabetic retinopathy (DR) risk in Chinese adult-onset T1DM, which integrates time-varying disease duration and HbA1c data. Using routine clinical indicators, this tool enables dynamic, quantitative assessment of DR risk during patient follow-up, providing a new approach for diabetes specialist nurses to deliver risk-stratified, individualized patient education and follow-up guidance. It has potential clinical value for implementing a proactive management model based on the DSMES framework—i.e., “risk assessment, dynamic education, shared decision-making”—and for improving patients’ DR awareness and adherence to screening.

## Data Availability

The raw data supporting the conclusions of this article will be made available by the authors, without undue reservation.
