# Supplementary material for: Construction of a dynamic prediction model for diabetic retinopathy risk in adults with adult-onset type 1 diabetes: a real-world longitudinal cohort study
Source: Front Clin Diabetes Healthc. 2026 Jul 28;7:1873754. doi: 10.3389/fcdhc.2026.1873754 (PMC13457143; doi:10.3389/fcdhc.2026.1873754)
Supplement: Supplementary file 1 [file Table1.docx]

Table S1. Comparison of longitudinal follow-up characteristics between patients with and without diabetic retinopathy (DR).

| **Characteristic** | **Non-DR group (n=358)** | **DR group (n=214)** | **P-value** |
| --- | --- | --- | --- |
| **Follow-up density** |  |  |  |
| Visit numbers |  |  | <0.001 |
| 2 visits | 221 (61.73%) | 106 (49.53%) |  |
| 3–4 visits | 99 (27.65%) | 91 (42.52%) |  |
| ≥5 visits | 38 (10.61%) | 17 (7.94%) |  |
| Median number of follow-up visits per patient | 2.3 (2.0, 3.0) | 3.0 (2.0, 5.0%) | <0.001 |
| **Time-related indicators** |  |  |  |
| Follow-up duration | 3.86 (2.01, 6.75) | 4.15 (2.13, 6.92) | 0.306 |
| Follow-up interval | 1.68 (1.12, 2.51) | 1.42 (0.95, 2.10) | 0.005 |
| Time to DR occurrence | — | 3.21 (1.58, 5.47) |  |
| **Time-varying covariates** |  |  |  |
| Dynamic disease duration | 5.15 (2.56, 8.21) | 12.07 (7.48, 15.85) | 0.029 |
| Time‑varying HbA1c | 8.62 ± 2.95 | 9.64 ± 3.04 | <0.001 |
| **HbA1c measurement interval** | 1.93 (1.40, 2.60) | 1.86 (1.35, 2.48) | 0.158 |
| **Distribution of HbA1c measurement intervals** |  |  | 0.597 |
| ≤1 quarter | 4 (1.12%) | 2 (0.93%) |  |
| 1–2 quarters | 297 (82.96%) | 177 (82.71%) |  |
| ≥3 quarters | 57 (15.92%) | 35 (16.36%) |  |

Data are expressed as the mean ± SD or the median (Q1, Q3) for continuous variables, where Q1 and Q3 denote the 25th and 75th percentiles, respectively; categorical variables are expressed as n (%).

① Visit numbers: number of valid fundus examinations from the date of first meeting inclusion criteria to the last follow-up or endpoint event (DR occurrence).

② Follow-up duration: time from study entry to last follow-up or endpoint event, used to balance follow-up completeness between groups.

③ Follow-up interval: time difference between two consecutive valid follow-up visits; the median value of all intervals for each patient was taken.

④ Time to DR occurrence: time from study entry to DR diagnosis.

⑤ Dynamic disease duration: cumulative disease duration at the endpoint of follow-up, calculated as (current clinical record date – diabetes diagnosis date)/365.25 (years).

⑥ Time‑varying HbA1c: reflecting the most recent glycemic control at each follow-up visit.

⑦ HbA1c measurement interval: time between two consecutive HbA1c measurements (unit: quarter).
